# Effect of Different Germination Conditions on Antioxidative Properties and Bioactive Compounds of Germinated Brown Rice

**DOI:** 10.1155/2015/608761

**Published:** 2015-03-12

**Authors:** You-Tung Lin, Cheng-Cheng Pao, Shwu-Tzy Wu, Chi-Yue Chang

**Affiliations:** ^1^Department of Bioindustry Technology, Dayeh University, Dacun, Changhua 51591, Taiwan; ^2^Department of Health Food, Chung Chou University of Science and Technology, Yuanlin, Changhua 51003, Taiwan

## Abstract

This study investigates antioxidative activity and bioactive compounds of ungerminated brown rice (UBR) and germinated brown rice (GBR). We used two rice cultivars (*Oryza sativa* L.), Taiwan Japonica 9 (TJ-9) and Taichung Indica 10 (TCI-10), as the materials in our experiments. The conditions for inducing germination are soaking time in water 24, 48, or 72 h; temperature 26 or 36°C; incubation in light or darkness; and open or closed vessels, in which the antioxidative activities and bioactive compounds of GBR were determined. We found that, in order to maximize antioxidative activity and bioactive compounds, germination should be under higher temperature (36°C), long soaking time (72 h), darkness, and closed vessel. GBR contains much higher levels of antioxidative activity and bioactive compounds than ungerminated brown rice (UBR). We found a strong correlation between antioxidative activities (DPPH radical scavenging ability, reducing power, and Trolox equivalent antioxidant capacity) and bioactive compounds (*γ*-oryzanols, tocopherol, and tocotrienol). Higher temperature (36°C) is also conducive to the production of GABA in GBR. These results are considered very useful research references for the development of future functional foods and additives.

## 1. Introduction 

Rice accounts for over 22% of global calories intake. More than 90% of the world's rice is grown and consumed in Asia [1]. Rice is classified according to the degrees of milling which differentiates brown rice from white rice. White rice is produced by mechanically removing bran from the surface of brown rice. During the process of germination by soaking brown rice in water, the chemical compositions of rice change drastically, because the biochemical activity produces essential compounds and energy, for the formation of seedling [2].

Antioxidants are thought to protect animal tissues from free radical-mediated degenerative diseases and aging. Rice bran contains high levels of several phytochemicals that promote antioxidative activities, as well as other reported health-beneficial properties [3]. The beneficial components of rice bran include sterols, *γ*-oryzanols, tocopherols, tocotrienols, and phenolic compounds [4]. Gamma-oryzanols, a mixture of lipophilic phytosterols that are composed of triterpene alcohols or sterols with ferulic acid ester, exhibit antioxidative properties and have cholesterol-lowering effect [5]. In addition to *γ*-oryzanols, tocopherols and tocotrienols in rice bran have also demonstrated beneficial effects such as antioxidative and antimutagenic activities [6] that play an important role in maintaining health. GBR is produced by soaking brown rice grains in water to promote germination, and *γ*-aminobutyric acid (GABA) is formed during this process [7]. Key bioactive compounds in GBR, for example, GABA, dietary fibre, ferulic acid, tocotrienols, magnesium, potassium, zinc, *γ*-oryzanol, and prolyl endopeptidase inhibitor, increase significantly in brown rice after germination [8]. When these bioactive compounds in GBR were benchmarked against those of milled ungerminated brown rice, they were 10 times greater in GABA, nearly four times greater in dietary fibre, vitamin E, niacin, and lysine, and three times greater in thiamine, pyridoxine, and magnesium [8]. GABA has several physiological properties such as neurotransmission and induction of hypotension and diuretic and tranquilizing functionalities [9–11]. GBR extracts contain GABA which can inhibit cancer cell from spreading [12].

The first part of this study aims to germinate brown rice under different sets of conditions: time, temperature, light, and vessel. Then we study the effect of different germination conditions on antioxidative properties and bioactive compound contents of GBR. Our objective is to measure compound contents and antioxidant properties of two domestic rice cultivars during grain imbibition and germination and to correlate them with the nutraceutical values of UBR.

## 2. Materials and Methods

### 2.1. Chemicals

Acetonitrile, ammonium acetate, ethyl acetate, isooctane (LC grade, purity 99%), *α*,*α*-diphenylpicrylhydrazyl (DPPH), butylated hydroxyanisole (BHA), *α*-tocophenol, GABA, *γ*-oryzanol, tocotrienol, 6-hydroxy-2,5,7,8-tetramethylchroman-2-carboxylic acid (Trolox), and 2,2′-azinobis-3-ethylbenzothiazoline-6-sulfonic acid (ABTS) were obtained from Sigma Chemical Co. (St. Louis, MO).

### 2.2. Preparation of GBR

Brown rice was provided by Taichung District Agricultural Research Station. We used two rice cultivars (*Oryza sativa* L.), Taiwan Japonica 9 (TJ-9) and Taichung Indica 10 (TCI-10), to prepare germinated brown rice (GBR). The following are the experimental conditions for inducing germination: soaking time 24, 48, or 72 h, temperature 26 or 36°C, incubation in continuous light or continuous darkness, and open or closed vessels, in which the antioxidative activities and bioactive compounds of GBR were determined.

### 2.3. Antioxidative Activity Assay

#### 2.3.1. Extraction of Samples

Different rice samples from different germination conditions were extracted by 70% ethanol (50 mg/mL, w/v) for a period of 24 hours and then filtrated and collected for the antioxidative activity assay.

#### 2.3.2. DPPH Radical Scavenging Activity

The radical scavenging activity was analyzed using the method by Shimada et al. [13]. Extracts (from Section 2.3.1) were mixed with 1 mL of 0.2 *μ*M DPPH methanol solution. The mixtures were then incubated at room temperature for 30 min before their absorbance was measured with a spectrophotometer (U-2800, IACHI, Japan) at 517 nm wavelength. BHA and *α*-tocophenol were the standard antioxidants we used to compare our experimental scavenging effect data against in this study. The formula for calculating scavenging effect is

(1) Scavenging  effect  % =1−sample  absorbance  valuewithout  sample−1     ·absorbance  value  of  a  control  group        without  sample−1×100%.

#### 2.3.3. Reducing Power

The reducing power was analyzed according to Oyaizu [14]. Two mL extract (from Section 2.3.1) was mixed with 0.5 mL of 0.2 M pH 6.6 phosphate buffer and 0.5 mL of 1% potassium ferricyanide solution, reacted in a water bath at 50°C for 20 min, and cooled in an ice bath for 3 min. The sample was mixed thoroughly with 0.5 mL of a 10% trichloroacetic acid solution and centrifuged. One mL of supernatant was collected and thoroughly mixed with 1 mL of deionized water and 0.2 mL of 0.1% ferric chloride solution. The mixture reacted in the dark at room temperature for 10 min and then the absorbance was measured with the spectrophotometer at the wavelength of 700 nm.

#### 2.3.4. Trolox Equivalent Antioxidant Capacity (TEAC)

The TEAC was analyzed according to Miller et al. [15]. Peroxidase 0.5 mL was mixed with 0.5 mL ABTS and 3.5 mL H_2_O_2_, to produce ABTS^•+^ radicals. After 6 min, it was mixed with 0.5 mL ethanol extract and then the absorbance was measured by the spectrophotometer at a wavelength of 700 nm. The data were calculated from the Trolox standard curve.

### 2.4. Determination of Bioactive Compounds

#### 2.4.1. GABA Analysis

We used a reversed phase high performance liquid chromatography (RP-HPLC) [16] to analyze GABA content in the rice samples. The samples were hydrolysed with concentrated HCl (12 N) before being analysed in HPLC. A linear gradient system was used with buffer A (0.1 M ammonium acetate, pH 6.5) and buffer B (0.1 M ammonium acetate containing acetonitrile and methanol, 44 : 46 : 10, v/v, pH 6.5) at 1 mL/min flowrate, with a C18 reversed phase column (Thermo C18, 4.6 × 250 mm, 5 *μ*m). The UV absorption detection was conducted at a wavelength of 254 nm.

#### 2.4.2. Gamma-Oryzanol Analysis

Gamma-oryzanol content was analyzed by the method of Chen and Bergman [3] with some modifications. Fifty mg of rice samples was extracted with 1 mL HPLC grade methanol for 1 min. The mixture was centrifuged for 10 min at 825 g and filtered (0.45 *μ*m) and analysed by RP-HPLC with the following mobile phase condition: methanol/acetonitrile/methane/acetic acid (50/44/3/3; v/v/v/v) at 1 mL/min flowrate, by using a C18 reversed phase column (Thermo C18, 4.6 × 250 mm, 5 *μ*m). The UV absorption detection was conducted at a wavelength of 330 nm.

#### 2.4.3. Tocopherol and Tocotrienol Analysis

The tocols were extracted and analysed by the method of Lin and Lai [17] with some modifications. 5 mL hexane was used to extract rice samples (250 mg) at 60°C for 30 min. The extracted mixture was then centrifuged at 3000 g for 10 min to collect its supernatant. The residue was extracted with hexane and centrifuged one more time. The supernatants collected from the two times of centrifuging were combined and analysed by HPLC. The mobile phase was 2.4% ethyl acetate in isooctane with a flowrate of 1.5 mL/min, by using a C18 reversed phase column (Thermo C18, 4.6 × 250 mm, 5 *μ*m). Detection was accomplished at fluorescence excitation wavelength of 290 nm and emission wavelength of 330 nm.

### 2.5. Statistics

In this study, each experiment was conducted in triplicate and the results were analyzed through a one-way analysis of variance using SAS (Statistic Analytic System) [18].

## 3. Results and Discussion

### 3.1. Nomenclature and Conduction of Experiments

**Figure 1:**
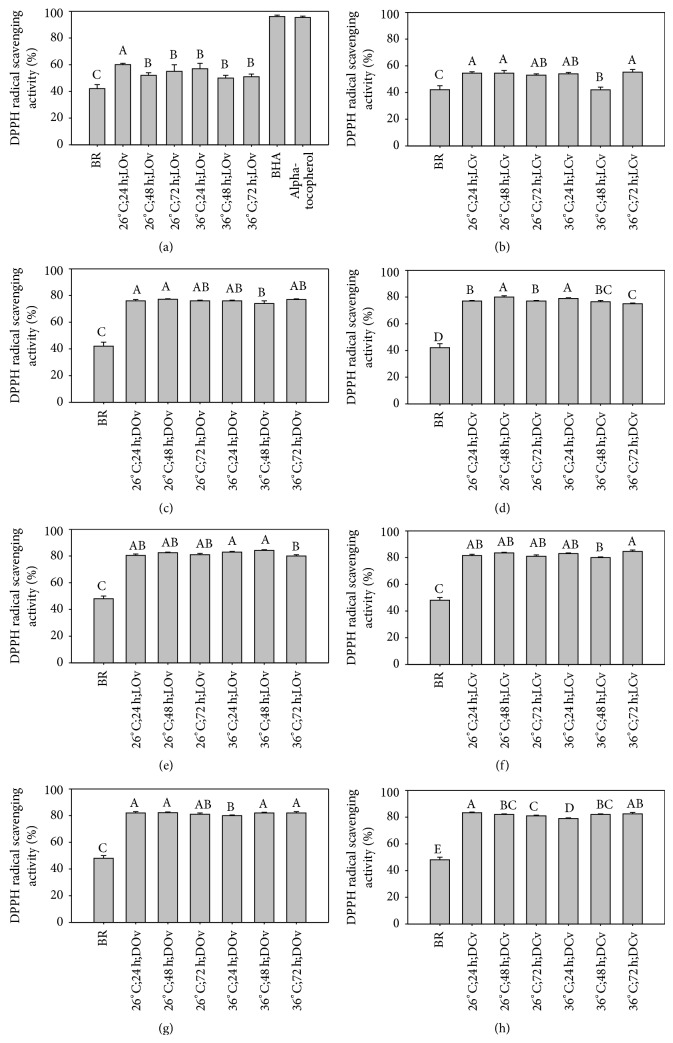
DPPH radical scavenging activity of brown rice germinated under different conditions. ((a)–(d)) TJ-9 brown rice. ((e)–(h)) TCI-10 brown rice. BR: ungerminated brown rice. A–E: data bars bearing identical letter are not significantly different (*P* < 0.05).

**Figure 2:**
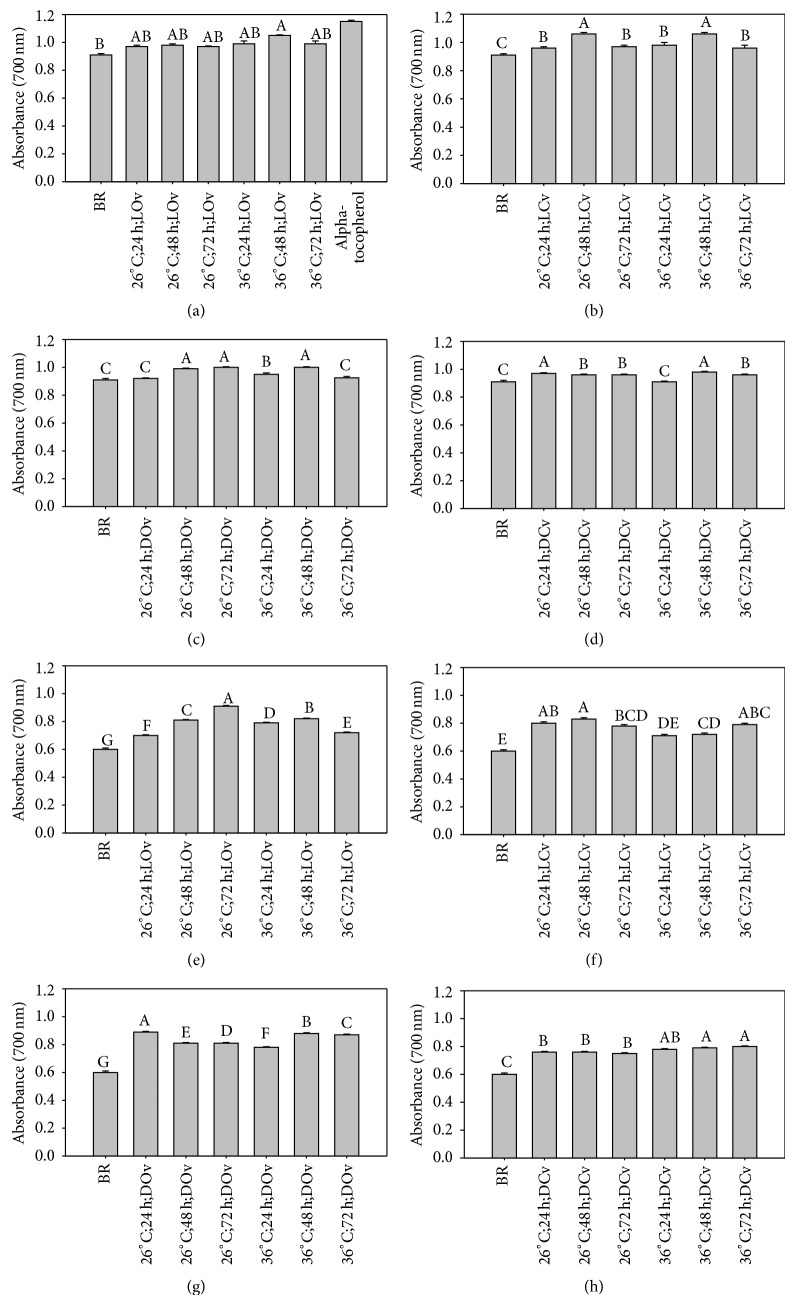
Reducing power of brown rice germinated under different conditions. ((a)–(d)) TJ-9 brown rice. ((e)–(h)) TCI-10 brown rice. BR: ungerminated brown rice. A–G: data bars bearing identical letter row are not significantly different (*P* < 0.05).

**Figure 3:**
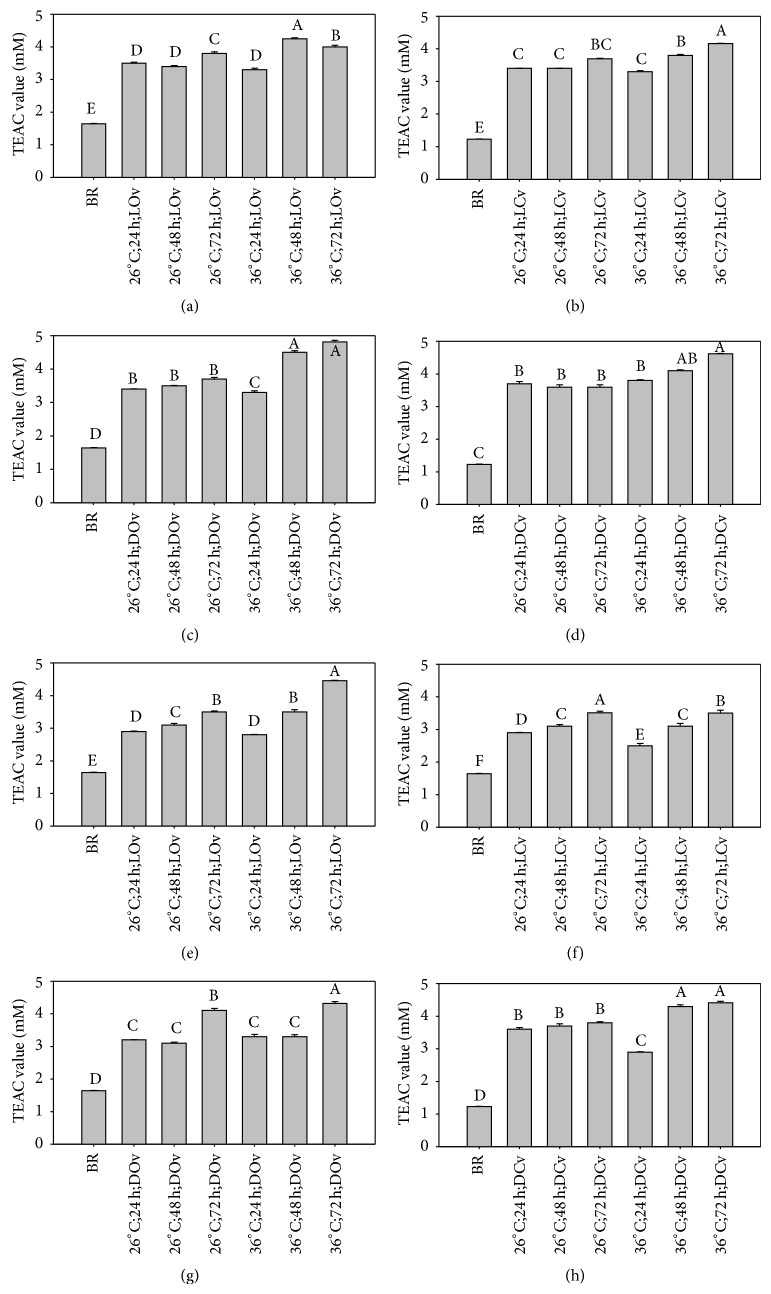
Trolox equivalent antioxidant capacity (TEAC) of brown rice germinated under different conditions. ((a)–(d)) TJ-9 brown rice. ((e)–(h)) TCI-10 brown rice. BR: ungerminated brown rice. A–F: data bars bearing identical letter row are not significantly different (*P* < 0.05).

Fifty samples were used in the experiments for measuring antioxidative activity (DPPH radical scavenging effects, reducing power, and TEAC). These 50 samples include two ungerminated brown rice (TJ-9 and TCI-10) and 48 germinated brown rice (TJ-9 and TCI-10 germinated under 24 different conditions) cultivars. As seen in Figures 1–3, 24 different conditions are denoted with the nomenclature as

(2) wC°; xh ;yz,

where *w*°C stands for germination temperature of either 26 or 36°C, *x*h stands for germination time of either 24, 48, or 72 h, *y* stands for germination lighting condition of either continuous light or continuous darkness, and *z* stands for germination vessel being either open or close. Therefore, an example can be given as 26°C;24 h;DOv that should be interpreted as under the germination condition of 26°C, 24 hours, in the dark and in an open vessel.

### 3.2. Antioxidative Activity

The DPPH radical scavenging effects of brown rice germinated under different conditions vary from 42.06% to 84.67% (Figure 1). As seen in Figure 1, the scavenging effects of ungerminated TJ-9 and TCI-10 are 42.06% and 48.06%, respectively. DPPH free radical scavenging ability can be enhanced through the germination process particularly in TCI-10 which was more impacted than in the case of TJ-9. However, DPPH free radical scavenging of both rice cultivars (both germinated and ungerminated) was less than that of BHT and *α*-tocopherol (96.13% and 95.36%), both synthetic antioxidants, at the same concentration. As rice extracts have a strong propensity to donate hydrogen atoms, the DPPH free radical scavenging might be caused by hydrogen donation ability [13]. In addition, TJ-9 data under dark germination show more effective DPPH free radical scavenging than those under light germination.

The reducing power of brown rice germinated under different conditions varies from 0.60 to 1.06 (Figure 2). As seen in this figure, ungerminated brown rice data of TJ-9 and TCI-10 were 0.91 and 0.60, respectively. Reducing power can be enhanced through germination particularly in TJ-9 which shows a stronger impact by germination than in TCI-10. The results demonstrate that some compounds of rice extracts are electron donors to free radicals which help to terminate or stabilize radical chain reactions; this agrees with the results by Chotimarkorn et al. [19].

Figure 3 shows the Trolox equivalent antioxidant capacity (TEAC) of brown rice germinated under different conditions which varies between 1.23 and 4.81 mM. The ungerminated brown rice data of TJ-9 and TCI-10 were 1.23 and 1.64 mM, respectively. The TEAC of rice extracts increases significantly with increasing germination time (*P* < 0.05). By definition, 1 mM of sample compound is equivalent to 2 mM TEAC.

**Figure 4:**
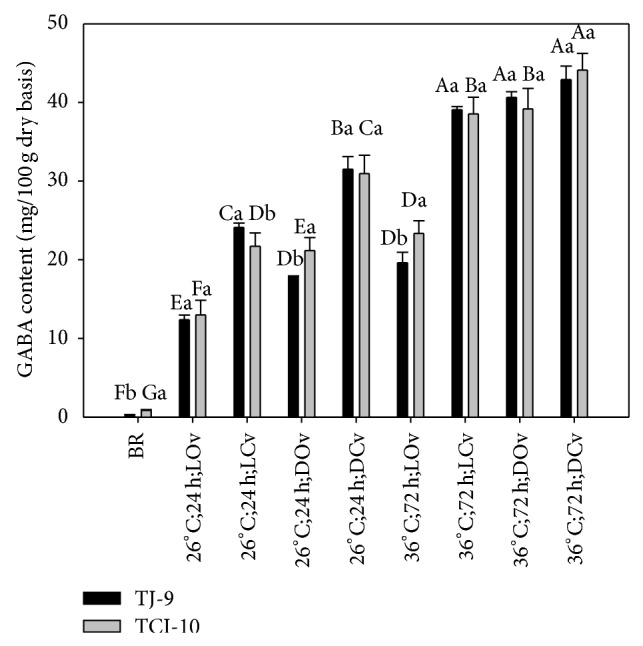
GABA contents of TJ-9 and TCI-10 brown rice germinated under different conditions. A–G: data bearing identical letter in the conditions are not significantly different (*P* < 0.05). a-b: data bearing identical letter in the cultivars are not significantly different (*P* < 0.05).

**Figure 5:**
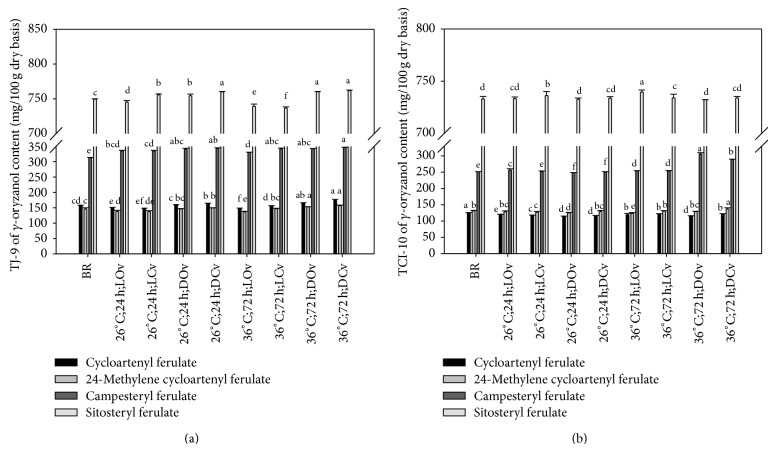
*γ*-Oryzanols contents of brown rice germinated under different conditions. a–f: data bearing identical letter in the conditions are not significantly different (*P* < 0.05).

**Figure 6:**
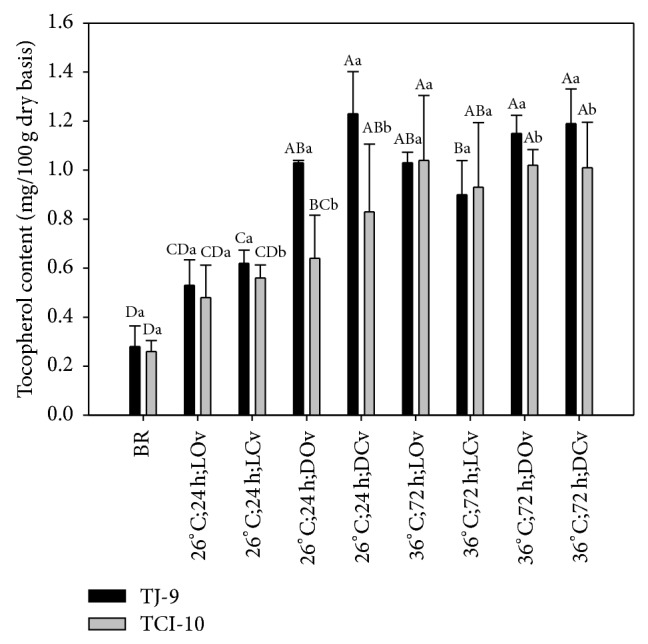
Tocopherol contents of TJ-9 and TCI-10 brown rice germinated under different conditions. A–D: data bearing identical letter in the conditions are not significantly different (*P* < 0.05). a-b: data bearing identical letter in the cultivars are not significantly different (*P* < 0.05).

**Figure 7:**
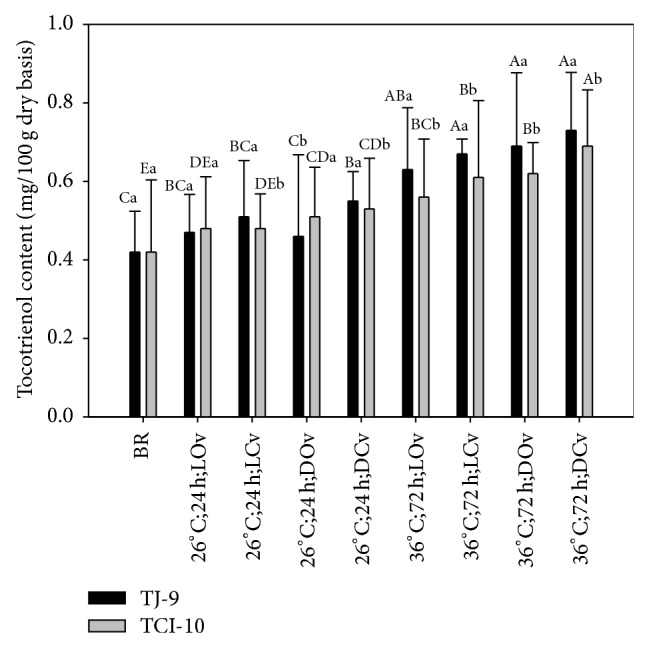
Tocotrienol contents of TJ-9 and TCI-10 brown rice germinated under different conditions. A–E: data bearing identical letter in the conditions are not significantly different (*P* < 0.05). a-b: data bearing identical letter in the cultivars are not significantly different (*P* < 0.05).

After we consolidated the antioxidative results from above, we pick out a set of samples for tests based on their antioxidative ability being on the high and low ends among all samples. As seen in Figures 4–7, these samples are created under the germination condition sets of either 26°C/36°C; 24 h/72 h; LOv/LCv, DOv/DCv. A total of 8 combinations of germination conditions were determined in order to investigate the effect of different antioxidative experimental variables.

### 3.3. The Content of GABA

The GABA contents of TJ-9 and TCI-10 brown rice germinated under different conditions are listed in Figure 4, which shows that the contents are affected by different germination and treatment processes. The GABA content of rice extracts increases significantly with increasing germination time (*P* < 0.05). In addition to the conditions of darkness and closed vessel, temperature is a key factor. The GABA contents of GBR range from 12.37 to 44.11 mg/100 g under different soaking conditions. A content of 44.11 mg/100 g was obtained under 36°C; 72 h; DCv which is in close proximity of those reported by Banchuen et al. [20], where the GABA contents of Niaw Dam Peuak Dam and Sangyod Phatthalung rice were 40.72 mg/100 g and 44.53 mg/100 g germinated under similar condition of 30°C, 48 h, and closed vessel. As reported by Komatsuzaki et al. [7], the increase in GABA content during water soaking may be due to the activation of glutamate decarboxylase (GAD), which converts glutamate to GABA at an optimal temperature of 40°C. This can explain why higher GABA is produced at 36°C than at 26°C. Steeping also leads to hypoxia due to the limited availability of oxygen in water for the grain [21] and GABA content may increase rapidly in plant tissues in response to hypoxia [22]. The above three studies agree with our results in Figure 4, where GABA content grows in a minimum case of 15 times from 0.79 mg/100 g of TJ-9 brown rice to 12.37 mg/100 g of 26°C; 24 h; LOv germinated TJ-9.

### 3.4. The Content of *γ*-Oryzanol

There are four main components of *γ*-oryzanols, namely, cycloartenyl ferulate, 24-methylene cycloartenyl ferulate, campesteryl ferulate, and sitosteryl ferulate [23]. They have also been shown to have antioxidative properties in many types of in vitro model systems [24, 25].  Figure 5 shows the *γ*-oryzanols contents of brown rice germinated under different conditions. As seen in the table, the highest *γ*-oryzanols contents appear under the conditions of 36°C; 72 h; D. The vessel open/close condition does not seem to be a factor.

### 3.5. The Content of Tocopherol and Tocotrienol

Figure 6 shows the contents of tocopherol in brown rice germinated under different conditions. In addition to the conditions of darkness and closed vessel, temperature and time are key factors in promoting the tocopherol contents. The tocopherol contents of GBR range from 0.48 to 1.23 mg/100 g, which is within the same range of the data reported by Moongngarm and Saetung [2], where the tocopherol content of RD-6 rice was 0.86 mg/100 g germinated under the condition of 30°C, 24 h.

Figure 7 shows the contents of tocotrienol in brown rice germinated under different conditions. The tocotrienol contents of GBR range from 0.42 to 0.73 mg/100 g, which is less than those reported by Pascual et al. [26], where the tocotrienol contents of SCS 114 Andosam, SCSBRS Tio Taka, and Epagri 109 rice were 1.87 mg/100 g, 2.26 mg/100 g, and 2.26 mg/100 g, respectively, germinated under the condition of 60°C, 6 h. We speculate the differences are caused by different cultivars and germinated conditions.

Also, in Figures 6 and 7, the contents of tocopherol and tocotrienol generated with germination of TJ-9 are higher than that of TCI-10 in general. This is in agreement with the result reported by Heinemann et al. [27] that the average content of vitamin E in* japonica* rice was higher than that in* indica* rice.

### 3.6. Correlations between Antioxidative Properties and Bioactive Compounds

Table 1 shows correlations among antioxidative properties and bioactive compounds of germinated brown rice (TJ-9 and TCI-10). For TJ-9, it shows all bioactive compounds except tocotrienol demonstrate strong correlations with DPPH radical scavenging ability (at level of *P* < 0.05). However, the correlation between reducing power and bioactive compounds is only found in *γ*-oryzanols; the correlation between TEAC and bioactive compounds is only found in tocopherol and tocotrienol (*P* < 0.05). For TCI-10, all three antioxidative activities have strong correlations with cycloartenyl ferulate, tocopherol, and tocotrienol. From these data, we can assert that *γ*-oryzanols and tocols both demonstrate good antioxidative abilities which is in agreement with previous studies [28, 29].

## 4. Conclusion

Germination by water soaking is a critical process in promoting the antioxidative activity and bioactive compounds in brown rice. GBR contains much higher levels of antioxidative activity and bioactive compounds than UBR. In order to maximize antioxidative activity and bioactive compounds, germination should be under the conditions of higher temperature (36°C in this study), long soaking time (72 h), darkness, and closed vessel. Also, higher temperature (36°C) during soaking process is a key factor in promoting GABA content in the GBR. Moreover, we find strong correlations between antioxidative activities (DPPH radical scavenging ability, TEAC, and reducing power) and bioactive compounds (*γ*-oryzanols, tocopherol and tocotrienol) in both cases of *P* < 0.05 and *P* < 0.01. These results are very useful research references for the development of future functional foods and additives and are also useful information to be used in the prevention of cancers.

## Figures and Tables

**Table 1:** Correlations among antioxidative properties and bioactive compounds of germinated brown rice.

Correlations	Cycloartenyl ferulate	24-Methylene cycloartenyl ferulate	Campesteryl ferulate	Sitosteryl ferulate	Tocopherol	Tocotrienol
TJ-9 germinated brown rice						
DPPH	0.65^**^	0.62^**^	0.81^**^	0.64^**^	0.74^**^	0.34
Reducing power	−0.45^*^	−0.47^*^	0.17	−0.49^**^	0.20	0.30
Trolox	0.29	0.31	0.83^**^	0.14	0.82^**^	0.83^**^

TCI-10 germinated brown rice						
DPPH	−0.71^**^	−0.14	0.19	0.20	0.68^**^	0.59^**^
Reducing power	−0.76^**^	−0.11	0.45^*^	−0.16	−0.53^**^	−0.53^**^
Trolox	−0.47^*^	−0.02	−0.46^*^	−0.27	0.77^**^	0.66^**^

^*^Correlation is significant at the level of *P* < 0.05.

^**^Correlation is significant at the level of *P* < 0.01.
